# Is preclinical diabetic retinopathy in diabetic nephropathy individuals more severe?

**DOI:** 10.3389/fendo.2023.1144257

**Published:** 2023-03-17

**Authors:** Hongyan Yao, Zijing Li

**Affiliations:** ^1^ Ningbo Eye Hospital, Ningbo University, Ningbo, China; ^2^ Department of Ophthalmology, Sun Yat-sen Memorial Hospital, Sun Yat-sen University, Guangzhou, China; ^3^ State Key Laboratory of Ophthalmology, Zhongshan Ophthalmic Center, Sun Yat-Sen University, Guangzhou, China

**Keywords:** preclinical diabetic retinopathy, diabetic nephropathy, optical coherence tomography angiography, estimated glomerular filtration rate, microstructural impairment

## Abstract

**Purpose:**

To analyse the retinal vessel density and thickness characteristics of diabetic nephropathy (DN) individuals with preclinical diabetic retinopathy (DR) using optical coherence tomography angiography (OCTA).

**Methods:**

This retrospective case−control study included 88 eyes of 88 type 2 DM patients with preclinical DR [44 non-DN (NDN) and 44 DN]. OCTA images and data were acquired using AngioVue 2.0 of the spectral domain OCT device. The foveal avascular zone (FAZ) area, superficial capillary plexus (SCP) and deep capillary plexus vessel densities, ganglion cell complex (GCC) and full retinal thicknesses, peripapillary capillary density and nerve fibre layer (RNFL) thickness were compared between the NDN and DN groups. The relationship between each renal function parameter and each OCTA parameter was analysed.

**Results:**

SCP vessel density, GCC thickness and full retinal thickness were significantly reduced in DN individuals compared to NDN individuals [(NDN versus DN) SCP vessel density: 46.65 ± 3.84% versus 44.35 ± 5.25%, p=0.030; GCC thickness: 100.79 ± 5.92 μm versus 93.28 ± 8.66 μm, p<0.001; full retinal thickness: whole area: 287.04 ± 13.62 μm versus 277.71 ± 15.10 μm, p=0.005). Within the peripapillary area, capillary density was also significantly reduced in the whole area (50.19 ± 3.10% versus 47.46 ± 5.93%, p=0.016) and some sectors in the DN group, though RNFL thickness was only decreased in some sectors. For all individuals, estimated glomerular filtration rate (eGFR) correlated significantly with most OCTA parameters and then showed a significantly negative correlation with FAZ area (β=−16.43, p=0.039) in multivariate linear regression analysis. In the NDN group, eGFR showed a significantly negative correlation with FAZ area (β=−18.746, p=0.048) and a significantly positive correlation with SCP vessel density (β=0.580, p=0.036).

**Conclusion:**

Preclinical DR may be more severe in DN individuals than in NDN individuals with regard to microvascular and microstructural impairment. Moreover, eGFR may be a good indicator for retinal microvascular impairment.

## Introduction

Diabetic retinopathy (DR), a common complication of diabetes mellitus (DM), remains the major cause of vision loss in the working-age population worldwide (1). Another common and severe complication of DM is diabetic nephropathy (DN), which may cause life-threatening end-stage renal disease (2). DR and DN are both microvascular DM complications, and they have similar pathophysiological mechanisms. Microvascular endothelial cells are regarded as common targets of hyperglycaemic impairment. Various common pathophysiological processes, including inflammation, oxidative stress and crosstalk between endothelial cells and pericytes/podocytes, exist in both complications (3). Previous clinical studies have suggested that the severity of DR parallels that of DN (4, 5).Zhang even found that DR might predict the renal function prognosis of type 2 DM (T2DM) patients with DN (5). Accordingly, in our routine clinical practice, severe DR, such as PDR and DME, is often detected in T2DM individuals with DN.

However, we also noticed that DN and DR are not consistent in some cases. Some DN individuals with poor renal function lack DR appearance in their fundus. DM patients without DR have been termed preclinical DR individuals in previous studies because reduced vessel density and other subtle microvascular lesions can be observed when using optical coherence tomography angiography (OCTA), a non-invasive cross-sectional real-time imaging technique, despite no obvious DR detected with conventional imaging methods (6, 7). Thus, we sought to determine whether more severe microvascular or microstructural alterations occur in DN than in NDN when using OCTA in preclinical DR individuals, which may help in further understanding the mutual effects and possible mechanisms between DR and DN. Unfortunately, few studies have focused on the retinal characteristics of DN individuals with preclinical DR.

Therefore, the purpose of this study was to analyse the retinal vessel density and thickness characteristics of DN individuals with preclinical DR using OCTA.

## Methods

### Subjects

This was a retrospective case−control clinical study. The study was conducted according to the principles of the Declaration of Helsinki and was approved by the institutional review board of Sun Yat-sen Memorial Hospital, Sun Yat-sen University (approval number: SYSEC-KY-KS-2021-263). Eighty-eight eyes of 88 T2DM patients with preclinical DR (44 NDN and 44 DN) were recruited from the Endocrinology Department between January 2018 and October 2021. The inclusion criteria included 1) a diagnosis of preclinical DR in the unilateral or bilateral eyes of T2DM patients; 2) age from 40 to 75 years; and 3) eyes with a logMAR best corrected visual acuity (BCVA) not more than 0.1. A random eye was chosen when the bilateral eyes involved preclinical DR. The diagnosis of T2DM, DR and DN were confirmed by an endocrinologist, an ophthalmologist and a nephrologist, respectively, based on criteria by the American Diabetes Association (8–10). The criteria for DN were 1) estimated glomerular filtration rate (eGFR) < 60 ml/min/1.73 m2, 2) urinary albumin to creatinine ratio > 30 mg/g for more than 3 months and 3) renal biopsy evidence in suspected patients. The values of eGFR were calculated using the Xiangya equation, a more accurate equation for eGFR in the Chinese population (11). Urinary albumin to creatinine ratio was tested in one random urine sample. A solid-phase fuorescent immunoassay was used to measure urinary albumin, and the Jafe rate method was applied to measure urinary creatinine (12). The exclusion criteria were as follows: 1) ocular diseases that may cause vision impairment, such as glaucoma, optic neuritis, uveitis and other retinal diseases; 2) spherical equivalent higher than −6 diopters or AL greater than 26 mm; 3) lens opacities affecting OCTA imaging; and 4) history of intraocular surgery.

The demographic and systemic data recorded for each individual included age, sex, body mass index (BMI), waist-to-hip ratio (WHR), haemoglobin A1c (HbAlc) levels, DM duration, DM therapy regimen, renal function (eGFR, blood urea, blood creatinine, blood uric acid, urinary protein and urinary microalbumin) and presence of hypertension. Hypertension was determined as >130/80 mmHg according to 2017 high blood pressure guidelines from the American College of Cardiology (13). Thorough ophthalmic examinations, including logMAR BCVA, intraocular pressure (IOP) (non-contact tonometer, Canon, Inc., Tokyo, Japan), axial length (AL), central anterior chamber depth (CACD), dilated fundus examination, colour fundus photos (Canon, Inc., Tokyo, Japan), OCTA (Optovue, Inc., Fremont, CA, USA) and FFA (if necessary and possible) (Microclear, Inc., Suzhou, China), were assessed in these individuals. AL and CACD were measured using IOLMaster (Carl Zeiss Meditec, Inc., Dublin, USA).

### OCTA image collection

OCTA images and data were acquired using AngioVue 2.0 of the spectral domain OCT device. Split-spectrum amplitude-decorrelation angiography was conducted to detect and analyse erythrocyte movement in vessels. An image of the 6 mm × 6 mm macular area and one of the 4.5 × 4.5 mm optic disc area were captured. Images with a scan quality < 6 were excluded. The vessel densities and retinal thickness of the macular and peripapillary capillary plexuses were then automatically exported. The recorded parameters included the foveal avascular zone (FAZ) area, superficial capillary plexus (SCP) and deep capillary plexus (DCP) vessel densities, ganglion cell complex (GCC) and full retinal thicknesses, peripapillary capillary density and nerve fibre layer (RNFL) thickness. Detailed retinal segmentations and divisions were similar to those in our previous work (14).

### Statistical analysis

Statistical analyses were performed using SPSS 26.0 (SPSS Inc. Chicago, IL, USA). Independent Student’s t tests were employed to compare normally distributed parameters, and Mann−Whitney tests were applied to compare nonnormally distributed parameters. Categorical variables were analysed using chi-squared tests. The relationship between each renal function parameter (level of eGFR, blood urea, blood creatinine, blood uric acid, urinary protein and urinary microalbumin) and each OCTA parameter (FAZ area, SCP and DCP vessel densities, GCC and full retinal thicknesses, peripapillary capillary density and RNFL thickness) was examined using bivariate correlation analysis in all individuals, the NDN group and the DN group, respectively. A value of p<0.05 was considered to be statistically significant. All OCTA parameters with statistical significance would be included in multivariate linear regression analyses adjusted for sex, age and DM duration for each renal function parameter. However, when OCTA parameters of different area (whole area, foveal, parafoveal or perifoveal) involved in the same terms (for example: SCP vessel density), only one representative parameter of this term [for example: SCP vessel density (whole area)] was included in multivariate analyses. Scatter diagrams were created using GraphPad Prism 7.0 (GraphPad Software, San Diego, CA, USA).

## Results

### Patient characteristics

Eighty-eight eyes of 88 T2DM patients with preclinical DR (44 NDN and 44 DN) were included in this study. Compared to the NDN group, the DN individuals had significantly increased WHR, longer DM duration, lower levels of eGFR, blood urea, blood creatinine, blood uric acid, urinary protein and urinary microalbumin and greater logMAR BCVA. A greater portion of individuals receiving subcutaneous insulin injection and a higher ratio of hypertension were noted in the DN group. No significant difference between the two groups was shown with regard to age, sex, BMI, HbAlc level or other variables. Details are shown in **Table 1**.

**Table 1:** Patient characteristics.

	NDN	DN	p
Patients (n)	44	44	NA
Age (years)	58.86 ± 11.60	59.80 ± 12.55	0.718^*^
Male:female (n)	30:16	30:16	1.000^#^
Anthropometrics			
BMI (kg/m^2^)	24.87 ± 3.18	29.42 ± 20.43	0.157^*^
WHR	0.91 ± 0.071	0.96 ± 0.073	0.004^*^
HbAlc (%)	8.96 ± 2.88	8.91 ± 1.89	0.930^*^
DM duration (years)	5.60 ± 5.70	7.07 ± 6.53	0.057
DM therapy regimen			
Oral medication (n, %)	32	14	**<0.001^#^**
Subcutaneous insulin injection (n, %)	2	10	
Both (n, %)	10	24	
Renal function			
eGFR (mL/min/1.73m^2^)	76.98 ± 6.70	65.09 ± 15.74	<0.001^*^
Blood urea (mmol/L)	5.42 ± 1.45	8.64 ± 5.19	<0.001^*^
Blood creatinine (μmol/L)	76.23 ± 10.01	123.81 ± 89.73	<0.001^*^
Blood uric acid (μmol/L)	344.84 ± 121.36	424.95 ± 153.16	0.014^*^
Urinary protein (g/L)	0.028 ± 0.027	0.37 ± 0.76	0.012^*^
Urinary microalbumin (mg/L)	13.46 ± 3.83	469.81 ± 1089.65	0.031^*^
Hypertension (n, %)	16, 36.36	32, 72.73	**<0.001^#^**
SBP (mmHg)	132.36 ± 13.87	138.18 ± 18.90	0.103^*^
DBP (mmHg)	79.77 ± 13.66	82.43 ± 15.04	0.388^*^
Ocular parameters			
BCVA (logMAR)	-0.011 ± 0.097	0.039 ± 0.078	0.009^*^
IOP (mmHg)	15.43 ± 2.11	15.59 ± 2.55	0.750^*^
CACD (mm)	3.17 ± 0.43	3.23 ± 0.29	0.549^*^
AL (mm)	23.83 ± 1.23	23.62 ± 0.70	0.395^*^
DR in fellow eye (n, %)	8	6	**0.560^#^**

NDN, non-diabetic nephropathy; DN, diabetic nephropathy; NA, not available; BMI, body mass index; WHR, waist-to-hip ratio; HbAlc, haemoglobin A1c; DM, diabetes mellitus; eGFR, estimated glomerular filtration rate; SBP, systolic blood pressure; DBP, diastolic blood pressure; BCVA, best corrected visual acuity; IOP, intraocular pressure; CACD, central anterior chamber depth; AL, axial length. *using Student’s t test; ^#^using chi-squared test; p value in bold style, p<0.05.

### OCTA findings

In the macular area, SCP vessel density was significantly reduced in the DN individuals compared to the NDN individuals (NDN versus DN: whole area: 46.65 ± 3.84% versus 44.35 ± 5.25%, p=0.030). Similar reductions were also observed in para- and perifoveal areas (parafoveal: 48.53 ± 4.54% versus 45.63 ± 7.62%, p=0.044; perifoveal: 47.26 ± 4.19% versus 44.86 ± 5.08%, p=0.025). However, there was no significant difference in FAZ or DCP vessel density between the two groups. Details are shown in **Table 2**. GCC and full retinal thicknesses were significantly decreased in the DN group (GCC thickness: whole area: 100.79 ± 5.92 μm versus 93.28 ± 8.66 μm, p<0.001; full retinal thickness: whole area: 287.04 ± 13.62 μm versus 277.71 ± 15.10 μm, p=0.005). Details are shown in **Table 3**.

**Table 2:** Vessel density in the macular area.

	NDN	DN	p
FAZ area (mm^2^)	0.30 ± 0.11	0.37 ± 0.26	0.198
SCP vessel density			
Whole area (%)	46.65 ± 3.84	44.35 ± 5.25	**0.030**
Foveal (%)	17.23 ± 7.19	15.48 ± 8.24	0.319
Parafoveal (%)	48.53 ± 4.54	45.63 ± 7.62	**0.044**
Temporal (%)	48.41 ± 5.82	46.43 ± 7.52	0.195
Superior (%)	49.06 ± 5.53	46.33 ± 7.88	0.079
Nasal (%)	47.97 ± 5.13	44.34 ± 9.93	**0.046**
Inferior (%)	48.67 ± 5.40	45.42 ± 8.94	0.055
Perifoveal (%)	47.26 ± 4.19	44.86 ± 5.08	**0.025**
Temporal (%)	42.50 ± 5.16	40.01 ± 7.15	0.08
Superior (%)	47.14 ± 4.70	45.34 ± 5.32	0.116
Nasal (%)	51.51 ± 3.86	48.72 ± 5.07	**0.007**
Inferior (%)	47.89 ± 4.27	45.46 ± 4.84	**0.022**
DCP vessel density			
Whole area (%)	46.29 ± 5.53	44.16 ± 7.32	0.148
Foveal (%)	32.86 ± 9.69	29.87 ± 9.82	0.176
Parafoveal (%)	52.13 ± 4.54	50.20 ± 6.93	0.148
Temporal (%)	53.29 ± 5.21	52.40 ± 6.82	0.517
Superior (%)	51.31 ± 5.41	50.43 ± 7.60	0.557
Nasal (%)	53.60 ± 5.33	50.36 ± 9.23	0.061
Inferior (%)	50.33 ± 5.75	47.62 ± 9.02	0.116
Perifoveal (%)	46.83 ± 6.08	44.53 ± 8.13	0.160
Temporal (%)	49.11 ± 6.43	46.12 ± 9.87	0.115
Superior (%)	46.16 ± 6.11	44.95 ± 8.34	0.466
Nasal (%)	46.15 ± 6.98	43.52 ± 8.87	0.149
Inferior (%)	45.87 ± 7.13	43.61 ± 8.16	0.198

NDN, non-diabetic nephropathy; DN, diabetic nephropathy; FAZ, foveal avascular zone; SCP, superficial capillary plexus; DCP, deep capillary plexus; p value in bold style, p<0.05.

**Table 3:** Retinal thickness in the macular area.

	NDN	DN	p
GCC thickness			
Whole area (μm)	100.79 ± 5.92	93.28 ± 8.66	**<0.001**
Foveal (μm)	52.38 ± 9.85	48.23 ± 11.11	0.081
Parafoveal (μm)	107.66 ± 8.67	98.50 ± 12.82	**<0.001**
Temporal (μm)	100.51 ± 7.94	93.52 ± 9.90	**<0.001**
Superior (μm)	110.26 ± 8.67	100.36 ± 14.55	**<0.001**
Nasal (μm)	108.50 ± 9.93	98.91 ± 13.30	**<0.001**
Inferior (μm)	111.38 ± 9.00	101.21 ± 16.43	**<0.001**
Perifoveal (μm)	100.55 ± 5.83	93.45 ± 7.99	**<0.001**
Temporal (μm)	86.29 ± 4.85	82.08 ± 6.20	**0.001**
Superior (μm)	100.47 ± 7.63	93.45 ± 10.38	**<0.001**
Nasal (μm)	117.09 ± 8.41	108.62 ± 11.00	**<0.001**
Inferior (μm)	98.40 ± 6.56	90.17 ± 10.68	**<0.001**
Full retinal thickness			
Whole area (μm)	287.04 ± 13.62	277.71 ± 15.10	**0.005**
Foveal (μm)	250.01 ± 24.65	243.24 ± 23.48	0.212
Parafoveal (μm)	319.53 ± 18.38	307.56 ± 19.54	**0.006**
Temporal (μm)	310.91 ± 18.27	301.17 ± 18.17	**0.019**
Superior (μm)	322.31 ± 18.66	311.89 ± 16.90	**0.011**
Nasal (μm)	324.72 ± 19.38	311.27 ± 21.50	**0.004**
Inferior (μm)	320.23 ± 18.47	305.89 ± 25.84	**0.006**
Perifoveal (μm)	278.74 ± 12.79	270.07 ± 14.76	**0.006**
Temporal (μm)	265.83 ± 13.13	259.97 ± 13.45	0.052
Superior (μm)	281.21 ± 13.81	274.44 ± 14.96	**0.039**
Nasal (μm)	298.62 ± 15.30	287.65 ± 20.88	**0.009**
Inferior (μm)	269.34 ± 12.47	258.46 ± 17.64	**0.002**

NDN, non-diabetic nephropathy; DN, diabetic nephropathy; GCC, ganglion cell complex; p value in bold style, p<0.05.

Within the peripapillary area, capillary density was also significantly reduced in the whole area (50.19 ± 3.10% versus 47.46 ± 5.93%, p=0.016) and some sectors in the DN group, but RNFL thickness was only decreased in some sectors (**Table 4**).

**Table 4:** Peripapillary capillary density and retinal nerve fibre layer (RNFL) thickness.

	NDN	DN	p
Peripapillary capillary density (%)			
Whole area (%)	50.19 ± 3.10	47.46 ± 5.93	**0.016**
Nasal superior (%)	46.82 ± 3.91	43.96 ± 7.42	**0.042**
Nasal inferior (%)	44.77 ± 4.55	43.08 ± 7.43	0.242
Inferior nasal (%)	47.68 ± 6.33	46.00 ± 7.00	0.277
Inferior temporal (%)	54.93 ± 5.13	51.66 ± 7.42	**0.030**
Temporal inferior (%)	51.27 ± 4.70	47.06 ± 6.95	**0.004**
Temporal superior (%)	54.87 ± 4.40	51.96 ± 6.77	**0.034**
Superior temporal (%)	51.65 ± 4.82	49.82 ± 7.93	0.233
Superior nasal (%)	48.94 ± 5.04	46.07 ± 7.15	**0.049**
Peripapillary RNFL thickness (μm)			
Whole area (μm)	112.14 ± 19.91	103.44 ± 21.46	0.072
Nasal superior (μm)	90.14 ± 13.30	91.73 ± 26.55	0.746
Nasal inferior (μm)	79.91 ± 13.80	82.04 ± 30.96	0.705
Inferior nasal (μm)	127.22 ± 23.74	125.53 ± 38.37	0.821
Inferior temporal (μm)	148.92 ± 26.74	129.91 ± 26.37	**0.003**
Temporal inferior (μm)	84.79 ± 66.18	65.62 ± 14.10	0.082
Temporal superior (μm)	97.08 ± 46.34	79.25 ± 15.19	**0.028**
Superior temporal (μm)	142.89 ± 17.68	131.00 ± 25.71	**0.023**
Superior nasal (μm)	128.04 ± 18.31	120.71 ± 37.78	0.294

NDN, non-diabetic nephropathy; DN, diabetic nephropathy; p value in bold style: p<0.05.

**Figure 1:**
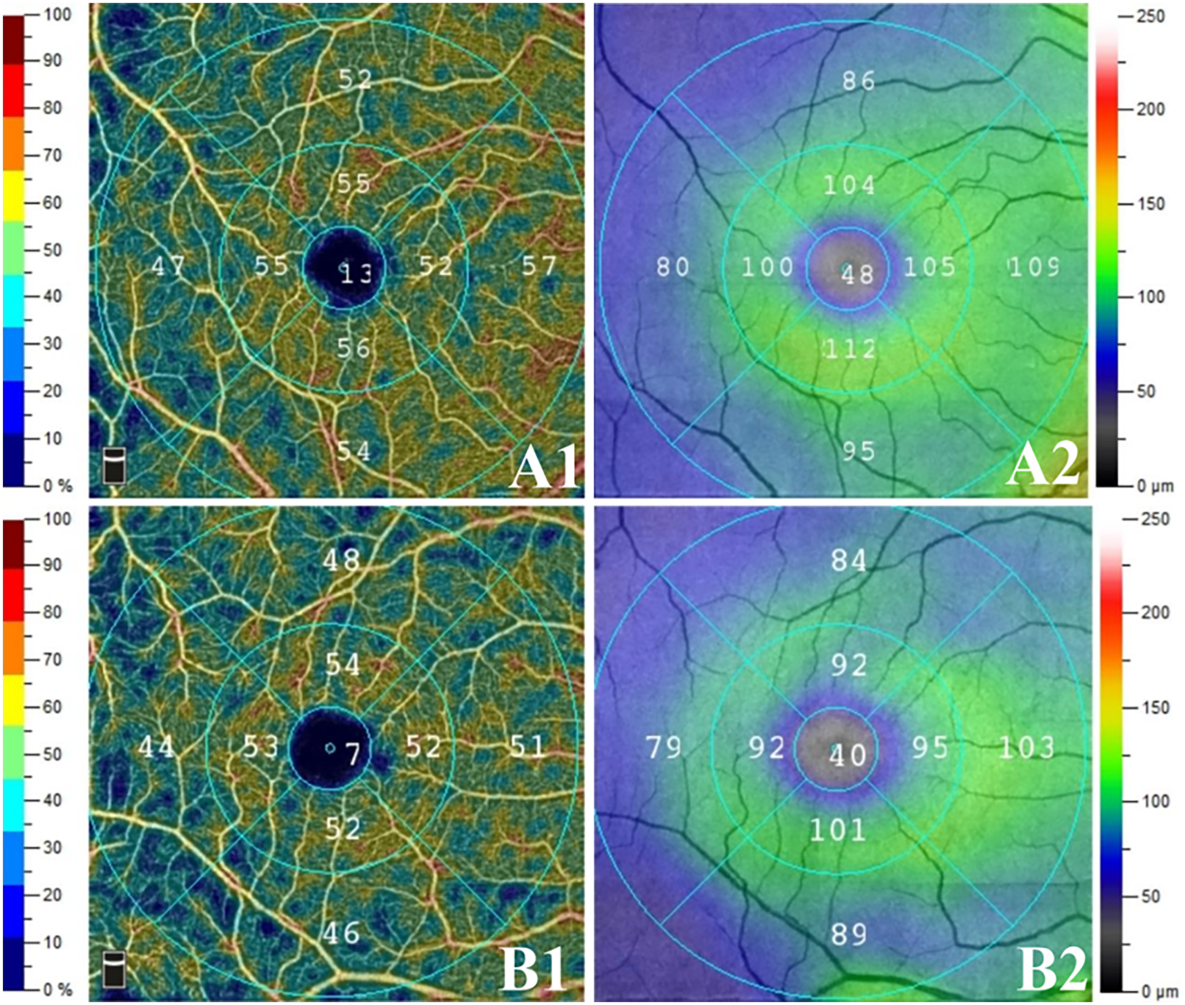
Representative images of the vessel density and GCC thickness in two groups. The right eyes of two age-matched male patients with preclinical diabetic retinopathy: **(A)** non- diabetic nephropathy (NDN) group, **(B)** DN group; (1) superficial capillary plexus (SCP) vessel density, (2) ganglion cell complex (GCC) thicknesses. Compared to NDN group, SCP vessel density and GCC thicknesses reduced in DN group. Moreover, more dark blue area was noticed in DN group than in NDN group.

Representative images of the vessel density and GCC thickness were shown in **Figure 1**.

### Relationship between renal function and OCTA findings

**Figure 2:**
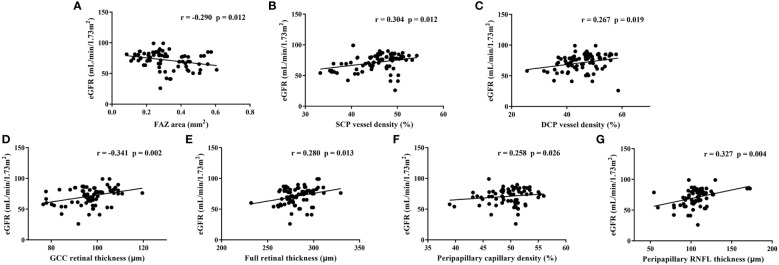
Relationships between estimated glomerular filtration rate (eGFR) and each representative optical coherence tomography angiography parameter in all individuals. FAZ: foveal avascular zone, SCP, superficial capillary plexus; DCP, deep capillary plexus; GCC, ganglion cell complex; RNFL, retinal nerve fibre layer; r, Pearson correlation coefficient.

For all individuals, only the level of eGFR correlated significantly with most of the OCTA parameters (**Table 5**), whereas there was no relationship between the level of the remaining renal function parameters (blood urea, blood creatinine, blood uric acid, urinary protein and urinary microalbumin) and OCTA parameters. Relationships between the level of eGFR and each representative OCTA parameter are shown in scatter diagrams (**Figure 2**). In multivariate linear regression analysis adjusted for sex, age and DM duration for eGFR, representative OCTA parameters with statistical significance including FAZ area, SCP and DCP vessel densities (whole area), GCC and full retinal thicknesses (whole area), peripapillary capillary density (whole area) and RNFL thickness (whole area) were included. Finally, the level of eGFR showed a significantly negative correlation with the FAZ area (β=−16.43, p=0.039) and a significantly positive correlation with the RNFL thickness (β=0.169, p=0.025).

**Table 5:** Correlation analysis between estimated glomerular filtration rate (eGFR) and optical coherence tomography angiography parameters in all individuals.

	eGFR (mL/min/1.73m^2^)	
	r	p
FAZ area (mm^2^)	-0.290	0.012
SCP vessel density		
Whole area (%)	0.304	**0.007**
Foveal (%)	0.241	**0.035**
Parafoveal (%)	0.259	**0.023**
Perifoveal (%)	0.330	**0.003**
DCP vessel density		
Whole area (%)	0.267	**0.019**
Foveal (%)	0.270	**0.017**
Parafoveal (%)	0.198	0.085
Perifoveal (%)	0.286	**0.021**
GCC retinal thickness		
Whole area (μm)	0.341	**0.002**
Foveal (μm)	0.278	**0.014**
Parafoveal (μm)	0.372	**<0.001**
Perifoveal (μm)	0.298	**0.008**
Full retinal thickness		
Whole area (μm)	0.280	**0.013**
Foveal (μm)	0.124	0.279
Parafoveal (μm)	0.285	**0.012**
Perifoveal (μm)	0.268	**0.018**
Peripapillary capillary density (μm)	0.258	0.026
Peripapillary RNFL thickness (μm)	0.327	0.004

FAZ, foveal avascular zone; SCP, superficial capillary plexus; DCP, deep capillary plexus; GCC, ganglion cell complex; RNFL, retinal nerve fibre layer; r, Pearson correlation coefficient; p value in bold style, p<0.05.

Relationships between renal function parameters and OCTA parameters for the NDN group are shown in **Table 6**. Representative OCTA parameters with significant correlations were then included in multivariate linear regression analyses adjusted for sex, age and DM duration for each renal function parameter. The multivariate linear regression analysis for eGFR included FAZ area, SCP vessel densities (whole area), DCP vessel densities (foveal), GCC thicknesses (parafoveal) and peripapillary capillary density (whole area), and finally showed that eGFR had a significantly negative correlation with the FAZ area (β=−18.746, p=0.048) and a significantly positive correlation with the SCP vessel density (β=0.580, p=0.036). FAZ area, SCP vessel densities (whole area) and GCC thicknesses (perifoveal) were included in the multivariate linear regression analysis for blood creatinine. The level of blood creatinine showed a significantly negative correlation with the SCP vessel density (β=-1.024, p=0.003) consequently. The multivariate linear regression analysis for urinary protein included SCP and DCP vessel densities (whole area), and finally showed that the level of urinary protein had a significantly positive correlation with the SCP vessel density (β=0.004, p=0.002). As for blood urea and urinary microalbumin, only one OCTA parameter was included in previous bivariate correlation analyses respectively. In linear regression analyses adjusted for sex, age and DM duration, a significantly positive correlation between the level of blood urea and the full retinal thickness (β=0.033, p=0.032) was shown, while no significant correlation was noticed between the level of urinary microalbumin and the OCTA parameter.

**Table 6:** Correlation analysis between renal function parameters and optical coherence tomography (angiography) parameters in non-diabetic nephropathy individuals.

	eGFR (mL/min/1.73m^2^)		Blood urea (mmol/L)		Blood creatinine (μmol/L)		Blood uric acid (μmol/L)		Urinary protein (g/L)		Urinary microalbumin (mg/L)	
	r	p	r	p	r	p	r	p	r	p	r	P
FAZ area (mm^2^)	-0.389	0.016	0.024	0.887	0.330	0.043	-0.097	0.584	-0.194	0.312	-0.222	0.286
SCP vessel density												
Whole area (%)	0.420	**0.008**	-0.074	0.655	-0.491	**0.002**	0.201	0.247	0.557	**0.002**	0.018	0.931
Foveal (%)	0.182	0.268	0.107	0.519	-0.318	**0.049**	0.026	0.884	0.257	0.178	0.036	0.863
Parafoveal (%)	0.509	**<0.001**	-0.066	0.691	-0.288	0.076	0.212	0.223	0.441	**0.017**	0.200	0.338
Perifoveal (%)	0.429	**0.006**	-0.109	0.508	-0.509	**0.001**	0.239	0.167	0.522	**0.004**	0.0003	0.998
DCP vessel density												
Whole area (%)	0.244	0.135	-0.253	0.12	-0.218	0.182	-0.028	0.873	0.368	**0.049**	-0.117	0.579
Foveal (%)	0.329	**0.041**	0.009	0.956	-0.304	0.06	0.060	0.732	0.295	0.12	0.166	0.427
Parafoveal (%)	0.112	0.496	-0.307	0.057	-0.199	0.224	-0.078	0.657	0.28	0.142	-0.157	0.453
Perifoveal (%)	0.291	0.072	-0.256	0.116	-0.232	0.156	0.010	0.955	0.339	0.072	-0.072	0.731
GCC retinal thickness												
Whole area (μm)	0.207	0.200	0.238	0.139	-0.265	0.099	-0.031	0.859	0.226	0.238	0.026	0.901
Foveal (μm)	0.18	0.265	0.097	0.552	-0.147	0.366	0.035	0.844	-0.017	0.932	-0.094	0.653
Parafoveal (μm)	0.419	**0.007**	0.278	0.083	-0.008	0.963	0.144	0.411	0.012	0.95	-0.006	0.978
Perifoveal (μm)	0.080	0.623	0.194	0.23	-0.346	**0.029**	-0.111	0.525	0.305	0.107	0.045	0.832
Full retinal thickness												
Whole area (μm)	0.181	0.264	0.339	**0.032**	-0.074	0.651	-0.090	0.609	0.067	0.729	-0.086	0.683
Foveal (μm)	0.116	0.477	0.249	0.121	-0.148	0.362	-0.023	0.895	-0.067	0.73	-0.409	**0.042**
Parafoveal (μm)	0.277	0.083	0.322	**0.043**	0.023	0.887	-0.019	0.912	-0.018	0.925	-0.206	0.323
Perifoveal (μm)	0.130	0.425	0.327	**0.039**	-0.104	0.524	-0.120	0.491	0.105	0.589	-0.009	0.965
Peripapillary capillary density (μm)	0.438	0.007	-0.217	0.204	0.035	0.840	0.100	0.580	0.138	0.500	-0.153	0.486
Peripapillary RNFL thickness (μm)	0.153	0.372	-0.12	0.484	-0.067	0.699	-0.037	0.839	0.256	0.206	0.368	0.084

eGFR, estimated glomerular filtration rate; FAZ, foveal avascular zone; SCP, superficial capillary plexus; DCP, deep capillary plexus; GCC, ganglion cell complex; RNFL, retinal nerve fibre layer; r, Pearson correlation coefficient; p value in bold style, p<0.05.

For the DN group, only the level of blood uric acid correlated positively with the perifoveal GCC thickness (r=0.359, p=0.043) and RNFL thickness (r=0.442, p=0.009). In multivariate linear regression analysis adjusted for sex, age and DM duration, the level of blood uric acid correlated positively with the RNFL thickness (β=2.692, p=0.009).

The corresponding details were summarized in **Table 7**.

**Table 7:** Included Optical coherence tomography angiography (OCTA) variables and variables with significance in multivariate linear regression analyses for different renal function variables.

Group	Renal function variable	Included OCTA variable in multivariate analysis	r	p	OCTA variable with significance in multivariate analysis*	β	p
All	eGFR	FAZ area	-0.29	0.012	FAZ area	-16.43	0.039
		SCP vessel density (whole area)	0.304	0.007	Peripapillary RNFL thickness	0.169	0.025
		DCP vessel density (whole area)	0.267	0.019			
		GCC retinal thickness (whole area)	0.341	0.002			
		Full retinal thickness (whole area)	0.28	0.013			
		Peripapillary capillary density	0.258	0.026			
		Peripapillary RNFL thickness	0.327	0.004			
							
NDN	eGFR	FAZ area	-0.389	0.016	FAZ area	-18.746	0.048
		SCP vessel density (whole area)	0.42	0.008	SCP vessel density (whole area)	0.58	0.036
		DCP vessel density (foveal)	0.329	0.041			
		GCC retinal thickness (parafoveal)	0.419	0.007			
		Peripapillary capillary density	0.438	0.007			
	Blood urea	Full retinal thickness (whole area)	0.339	0.032	Full retinal thickness (whole area)	0.033	0.032
	Blood creatinine	FAZ area	0.33	0.043	SCP vessel density (whole area)	-1.024	0.003
		SCP vessel density (whole area)	-0.491	0.002			
		GCC retinal thickness (perifoveal)	-0.346	0.029			
	Urinary protein	SCP vessel density (whole area)	0.557	0.002	SCP vessel density (whole area)	0.004	0.002
		DCP vessel density (whole area)	0.368	0.049			
	Urinary microalbumin	Full retinal thickness (foveal)	-0.409	0.042	NA		
							
DN	Blood uric acid	GCC retinal thickness (perifoveal)	0.359	0.043	Peripapillary RNFL thickness	2.692	0.009
		Peripapillary RNFL thickness	0.442	0.009			

The included OCTA variables in multivariate linear regression analyses were those variables with statistical significance in previous univariate analyses. eGFR, estimated glomerular filtration rate; FAZ, foveal avascular zone; SCP, superficial capillary plexus; RNFL, retinal nerve fibre layer; DCP, deep capillary plexus; GCC, ganglion cell complex; NDN, non- diabetic nephropathy; DN, diabetic nephropathy; r, Pearson correlation coefficient; β, regression coefficient; ^*^adjusted for sex; age and DM duration.

## Discussion

Is preclinical DR in DN individuals more severe? According to our present study, the answer may be “yes” when compared to the NDN individuals.

In our study, SCP vessel density rather than DCP vessel density was significantly reduced in DN individuals. Zhuang’s study revealed that SCP and DCP vessel densities decrease as chronic kidney disease (CKD) progresses in DM patients but that SCP vessel density reduction is more pronounced (15). Wang’s study showed that DN patients had reduced macular vessel density compared to NDN patients, but macular vessel density was not divided into the SCP and DCP (16). Our result was somewhat consistent with these studies. Nevertheless, Zhuang’s and Wang’s studies included preclinical DR and DR simultaneously, and the percentage of DR in the DN group was obviously higher than that in NDN. Vessel density may therefore be greatly affected by the constitution of DR at different stages. Our study focused specifically on preclinical DR, which may minimize the above effects and for the first time provide a possible answer to the interesting question: Is preclinical DR in DN individuals more severe? As stated in previous studies, capillary impairment is more remarkable in the DCP than in the SCP in preclinical and nonproliferative DR as the disease develops (17, 18). Histologically, the SCP is a network supplying and connecting other capillary plexuses, whereas the DCP is composed of lobular configurations without abundant capillary connections (19). The SCP may have stronger self-regulatory ability than the DCP. In this sense, the DCP may be more vulnerable than the SCP and may serve as an initial marker in early-stage DR. However, our study revealed that SCP vessels seem to reduce more obviously than DCP vessels in DN individuals, which was quite an interesting finding. This may suggest that DN patients have more pronounced and advanced capillary dropout than NDN patients, even when they only have “preclinical DR”. The explanation for such advanced capillary dropout may be attributed to the complicated mechanisms of DN and its complications. The renin-angiotensin-aldosterone system exerts a strong vasoconstrictive effect and possibly induces haemodynamic disturbances in the retinal microvasculature (20). Oxidative stress and inflammation occurring in DN might also accelerate the process of retinal vascular endothelial cell impairment (21). Furthermore, anaemia in DN secondary to erythropoietin reduction, uraemic toxin accumulation and dialysis might be responsible for capillary impairment, as stated in previous studies (22, 23).

Another interesting finding in our study was that the peripapillary capillary density was reduced in DN. The radial peripapillary capillary plexus (RPCP) and the SCP are closely related and components of the superficial vascular complex. The RPCP is nourished by precapillary arterioles from the SCP and is driven by postcapillary venules into the SCP (19). Hence, it may be understandable that SCP vessel density and peripapillary capillary density simultaneously decrease. However, peripapillary capillary data were recorded based on limited resources, and the standard was different; thus, comparisons cannot be fully performed. Cankurtaran’s study suggested that preclinical DR patients with microalbuminuria have a higher peripapillary capillary density than those with normal albuminuria, which was similar to our result (24). The major difference was that the population was classified based on the level of albuminuria in Cankurtaran’s study but that patients were classified based on the diagnosis of DN in our study.

Compared to capillary density, a more pronounced alteration in DN individuals was reduced macular retinal thickness. This was a novel finding, as no previous study has reported similar results. Macular retinal thickness was not altered as CKD or albuminuria progressed in previous DN-related studies (15, 24). One possible explanation for the difference between these results is the DR stage variation in the included population, as stated above. Another possible explanation is the controversial mechanism of DN. Classically speaking, proteinuria is followed by decline in renal function. However, some DM patients have progressive reduction in eGFR with microalbuminuria regression or even without proteinuria, which may suggest that alteration of eGFR occurs independently of the presence of albuminuria (25, 26). Microalbuminuria and decreased eGFR can be present simultaneously or separately in DN. This may pose a challenge for the definition of DN, as an abnormal level of eGFR or urinary albumin may be regarded as DN. The group division standard is not uniform in all studies. According to our results, we speculate that the diffuse shrinkage of retinal thickness observed was secondary to capillary dropout. Uraemic toxins might also be a crucial factor. A recent study showed that accumulation of uraemic toxins such as parathyroid hormone and β2-microglobulin correlates closely with GCC impairment in CKD patients without diabetes or dialysis (27). Moreover, dialysis is a potential reason. A Japanese study revealed that haemodialysis not only contributes to relieving DME but also helps to decrease retinal thickness after a one-year follow-up (28). However, this study mainly included DME patients rather than preclinical DR, the mechanism might be partially different in these individuals. Haemodialysis may to some degree accelerate the flow of the retinal/subretinal fluid as well as eliminate toxins and inflammatory particles (29).

For all individuals, eGFR was closely related to most OCTA parameters. Even after multivariate linear regression analysis adjusted for age and sex, the level of eGFR still showed a significantly negative correlation with the FAZ area. In the NDN group, the level of eGFR was associated with the FAZ/SCP vessel density. These results in our study suggest that eGFR may be a good indicator for microvascular impairment in all preclinical DR individuals and NDN individuals. Similar to our study, Vadalà’s and Wang’s studies showed that eGRF correlated positively with macular vessel density in T2DM patients with CDK (16, 30). In contrast, no association between eGFR and FAZ area/vessel density in DM patients was reported in Cheung’s study, while this study showed that large intercapillary area is associated with eGFR (31).These contradictions among studies may be related to a variation in DR stage, a different adoption of the eGFR equation, various measurement methods of OCTA parameters and a relatively small sample. In addition, it may be affected by complicated systemic confounding factors among different study groups. In the NDN group, the urinary protein level was positively associated with the SCP vessel density, but no association between the urinary microalbumin level and each OCTA parameter was noted. This finding is at odds with some publications. In a Turkish study, urinary microalbumin levels correlated negatively with DCP vessel density in T2DM patients with and without DN (32). Yao’s study revealed that macular vessel density correlated negatively with urinary protein levels in adults with primary nephrotic syndrome (33). The analysed subgroup in our study had NDN rather than NDN combined with DN or other kinds of nephropathy, and thus the differences among individuals may be indistinctive. Moreover, in the NDN group, proteinuria may be physiological or very mild, and its correlation with OCTA parameters might be less meaningful. In the DN group, most OCTA parameters lacked correlation with renal function parameters. A potential explanation for this is that the process of DN had actually initiated and progressively worsened the retinal microvasculature when DN was not diagnosed in preclinical DR individuals. At this stage, renal function and OCTA parameters change in parallel. However, as DN develops, retinal impairment may not only be affected by renal functions but also be influenced by secondary systemic alterations, such as electrolyte imbalance, anaemia, hypertension, uraemic toxin accumulation and dialysis (34). These factors may cause an unparallel relationship between OCTA parameters and renal function parameters in the DN group.

We noticed that NDN group has better BCVA when compared to DN group. Cataract severity may mainly account for the variation. In addition, DM duration seemed to be longer in DN group than in NDN group. Though the difference didn’t reach statistical significance, the effect of DM duration should be considered. However, the subsequent multivariate linear regression analyses adjusted for sex, age and DM duration may partially avoid the effect of DM duration.

The major limitation of our study was the small sample and its retrospective nature. Another limitation was that the included individuals were Chinese, which cannot represent the entire population of T2DM individuals. The topic deserves further exploration using a well-designed prospective study with a large sample size.

In conclusion, in preclinical DR, DN patients have reduced retinal vessel density and thickness compared to NDN patients. Preclinical DR may be more severe in DN individuals than in NDN individuals with regard to microvascular and microstructural impairment. Moreover, eGFR may be a good indicator for retinal microvascular impairment.

## Data availability statement

The original contributions presented in the study are included in the article/supplementary material. Further inquiries can be directed to the corresponding author.

## Ethics statement

The studies involving human participants were reviewed and approved by Sun Yat-sen Memorial Hospital, Sun Yat-sen University. Written informed consent for participation was not required for this study in accordance with the national legislation and the institutional requirements.

## Author contributions

All authors contributed to the study conception and design. Material preparation, data collection and analysis were performed by ZL and HY. The first draft of the manuscript was written by ZL and HY, and all authors commented on previous versions of the manuscript. All authors contributed to the article and approved the submitted version.
